# Interactive synchrony and infants’ vagal tone as an index of emotion regulation: associations within each mother- and father-infant dyad and across dyads

**DOI:** 10.3389/fpsyg.2023.1299041

**Published:** 2023-12-19

**Authors:** Nilo Puglisi, Nicolas Favez, Valentine Rattaz, Manuella Epiney, Chantal Razurel, Hervé Tissot

**Affiliations:** ^1^Faculty of Psychology and Educational Sciences, University of Geneva, Geneva, Switzerland; ^2^Center for Family Studies, Department of Psychiatry, Lausanne University Hospital and University of Lausanne, Lausanne, Switzerland; ^3^Department of Obstetrics and Gynecology. University of Geneva Hospital, Geneva, Switzerland; ^4^Department of Midwifery, University of Applied Sciences Western Switzerland, Geneva, Switzerland

**Keywords:** parent-infant interactions, emotion regulation, synchrony, vagal tone, infancy

## Abstract

**Introduction:**

Studies have shown that infants’ emotion regulation capacities are closely linked to the quality of parent-infant interactions. However, these links have been mostly studied in mother-infant dyads and less is known about how the quality of father-infant interactions contributes to the development of emotion regulation during infancy. In this study, we aimed to investigate the links between interactive synchrony (i.e., an index of the quality of parent-infant coordination of interactive behaviors) and infants’ vagal tone (i.e., a physiological index of emotion regulation). To understand the respective contributions of both parents, as well as the interrelations between the functioning of both dyads within a family, we observed mothers and fathers from 84 families interacting with their infants.

**Methods:**

Synchrony was assessed by using the CARE-Index; infants’ vagal tone was derived from the analysis of infants’ electrocardiograms recorded during the interactions. Moreover, to take the play’s order into account, we counterbalanced the procedure, so that approximately half of the mothers played first. We specified a first structural equation modeling (SEM) model to investigate the associations between interactive synchrony and the infants’ root mean square of successive differences (RMSSD), an index of vagal regulation, in the two successive parts of the play. We conducted a multigroup analysis in a second SEM model to investigate the associations of the first SEM model in two groups based on the order of interaction.

**Results:**

The results of the SEM models showed that greater synchrony was related to greater infant RMSSD within mother-infant dyads and across one dyad to the other dyad in the full sample and in the group of fathers who interacted first with the infants. The associations between synchrony and infant vagal tone within father-infant dyads never appeared to be significant, nor did any associations within each dyad and across dyads when mothers interacted first.

**Discussion:**

This study highlights that the links between interactions and infants’ vagal tone are sensitive to family members’ interdependencies and some conditions (the order of interaction).

## 1 Introduction

### 1.1 The interpersonal component of emotion regulation

The interpersonal component of emotion regulation is crucial in early infancy because, by interacting with parents, immature infants develop emotion regulation patterns that allow them to be progressively autonomous in recognizing their internal states and regulating emotions (Sameroff, 2004). Emotion regulation can be defined as a “process responsible for monitoring, evaluating and modifying emotional reactions, especially their intensive and temporal features, to accomplish one’s goal” (Thompson, 1994, p. 27). This definition reflects a functionalist view of emotion regulation, according to which emotion regulation allows the infant to achieve goals in the surrounding environment (e.g., for the infant to be fed, comforted, or protected; Campos et al., 1994; Cole et al., 1994). Emotion regulation involves intrinsic and extrinsic processes within the individual, with a strong influence from and on the environment. At 3 months, the quality of early interactions with the parents, the infant’s main social interactants, helps the infant to shape behavioral and physiological patterns of emotion regulation, with consequences for the infant’s socioemotional development (Tronick and Cohn, 1989; Cole et al., 2004; Cabrera et al., 2014; Morris et al., 2017; Low et al., 2019).

One index of the quality of parent-infant interactions that has been previously linked with emotion regulation is interactive synchrony, which reflects the quality of the mutual regulation of the interaction by the parent and the infant (Tronick, 2007; Bernard et al., 2013; Nguyen et al., 2020). Provenzi et al. (2018) defined interactive synchrony as the “degree of congruence between trans-modal behaviors of two partners, which is lagged in time and which promotes infants’ learning of emotional regulation skills and the emergence of expectations on interactive repertoires” (p. 12). Interactive synchrony is linked with better cognitive development, fewer externalizing and internalizing symptoms, and adaptive self-regulation, with effect sizes ranging from small to large (e.g., Laible and Thompson, 2000; Kochanska et al., 2008; Feldman and Eidelman, 2009; Pesonen et al., 2010; Hinnant et al., 2013; Suveg et al., 2016). Interactive synchrony implies that the parent and the infant exchange and coordinate behavioral (e.g., gaze, affection, voice, and touch) and physiological (e.g., brain networks, affiliative hormones, and autonomic responses) signals, within each other, between each other, and between the physiology of one member of the dyad and the behavior of the other member (Tronick and Cohn, 1989; Feldman et al., 2010; Beebe et al., 2016; Provenzi et al., 2018). The repeated experience of synchronous exchanges during parent-infant interactions fosters the emergence of repetitive and rhythmic matched patterns within the dyad characterized by being “concurrent” (when the parent is happy, the infant is happy) and “sequential” (variations in the parent predict variations in the infant) between partners (Feldman, 2007a; Wass et al., 2020). During the perinatal period, synchrony is predominantly driven by the parent, who through direct glances, expressions of positive affect, vocalizations, and affectionate touch coordinates with the infants’ attention during awake time. Later in infancy, the infant becomes an active social partner capable of co-constructing interactive synchrony with the parent(s) through the active coordination of gaze, affective expressions, co-vocalizations, and touch patterns (Feldman, 2007b, 2015). Although being in synchrony is desirable, synchrony in social interactions is most often difficult to achieve. The reason is that social interactions naturally contain mistakes or external perturbations and thus many possibilities for moments of miscoordination that reduce interactive synchrony (Markova and Nguyen, 2022). For example, moments of miscoordination can occur when a parent misunderstands the infant’s signals by being withdrawn when the infant is willing to interact or by trying to engage the baby when he or she is fussy. However, despite moments of miscoordination, both partners may maintain a certain degree of interactive synchrony when they implement behavioral and physiological changes appropriate to the signals coming from the other partner (Tronick and Gianino, 1986; Beeghly et al., 2011). For example, a withdrawn parent may stay involved in the interaction by making eye contact with the infant, or a parent with a fussy baby might gently pat the baby’s hand to distract and maybe soothe the baby, avoiding overstimulating activities.

Interactive synchrony involves regulatory behaviors, observable during an interaction, and emotion regulation processes, measurable on a physiological level through vagal tone. Vagal tone is a valid index of physiological regulation; it reflects the vagus nerve’s contribution to the autonomic nervous system mechanisms related to emotion regulation. According to Porges (2011, 2021) polyvagal theory, vagal tone variations relate to the experience and expression of social, emotional, and communicative regulation during interactions. High vagal tone in early childhood has been associated with better regulation and fewer externalizing, internalizing, and cognitive problems across development. Conversely, low vagal tone has been shown to correlate with difficulty in regulation, poorer sustained attention, more impulsiveness, and greater disinhibition (Feldman, 2006; Graziano and Derefinko, 2013; Provenzi et al., 2015; Wagner et al., 2021). The suppression of vagal tone is a physiological indication of difficulty in social regulation and emotion regulation processes. The study of vagal tone in parent-infant interactions has shown, predominantly in the mother-infant dyad, that physiological variations can be observed depending on the quality of the interactions (Moore and Calkins, 2004; Lunkenheimer et al., 2020). When the quality of the interaction is high, with adaptive coregulation between parent and infant behavior, the infant vagal tone generally increases to support behavioral organization during social involvement (e.g., gaze sharing, shared attention). Conversely, when the quality of the interaction decreases, the lower coregulation generates a stress for the infant and is associated with lower vagal tone to support behavioral responses to a difficult interaction (e.g., avoidance of adult gaze, crying; Feldman et al., 2010; Provenzi et al., 2015). Pratt et al. (2015) found that mother-infant synchrony positively correlated with and predicted vagal withdrawal. In addition, mother-infant synchrony may strengthen vagal regulation in infants with high and low negative reactivity. Provenzi et al. (2015) observed a higher frequency of dyadic matching of affective states and dyadic repair in dyads with optimal vagal functioning. To summarize, infant’s vagal tone is a crucial aspect to consider when investigating the interpersonal aspect of emotion regulation development.

### 1.2 The interdependencies between mother- and father-infant interactions

During infancy, different adults (e.g., parents, grandparents, aunts, uncles, and professional caregivers) may shape social interactions with infants, thus contributing to the development of regulation patterns (Bronfenbrenner, 1974; Kokkinaki et al., 2012; Kokkinaki and Pratikaki, 2014). However, the most frequent interactions for infants occur with primary caregivers, that is, one or both parents, as they are closest to them and quickly provide them with the care they need to survive. Interactions with each parent allow the infant to experience different types of interactive synchrony, with different consequences on the coordination of physiological states and interactive behavior within each dyad (Lamb et al., 1987; Moore and Calkins, 2004; Skibo et al., 2020; Wu and Feng, 2020; Rodrigues et al., 2021). For example, during interactions with fathers, which often focus on highly stimulating physical play, interactive synchrony tends to involve the regulation of higher levels of positive arousal than it does during interactions with the mother, which often focus on the regulation of mutual gazes and vocalizations during face-to-face interactions (Feldman, 2003). In Western societies, mothers still mostly play the role of primary caregiver in early infancy, and thus previous research on the interpersonal components of emotion regulation in early infancy has largely focused on the mother-infant dyad. Furthermore, previous research has often considered the mother-infant dyad without taking into account other social interactions that might influence it (father-infant dyad) or encompass it (the whole family). Family system theorists, however, have long suggested that consideration of the connections between the different members of a family and their influence on the infants’ functioning is necessary for a more accurate view of family influences on infant development. According to family system theory (Minuchin, 1974, 1985; Cox and Paley, 1997), the family system is composed of several subsystems, each of which has specific properties and the potential to influence and be influenced by the others. Minuchin (1985) argued that each subsystem can only be accurately understood in the context of its relationships with the others, as subsystems do not function in isolation from one another. What happens in one dyad (mother-infant) is likely to influence and be influenced by what happens in another dyad (father-infant). In sum, the infant’s interactions with the mother and father are non-independent because complex interdependencies exist in a family.

The interdependencies in a family may function cumulatively during parent-infant interactions, so that adaptive or maladaptive functioning in one subsystem (e.g., parent-infant) spreads to other subsystems (e.g., interparental), leading to multiple factors influencing the infant’s emotion regulation. However, it is also possible that several subsystems may compensate for others. Thus, the maladaptive effect of one subsystem on the infant’s emotion regulation may be compensated by the protective effect of another system. Examples of interdependencies in a family are the spillover effect and the crossover effect. The spillover effect refers to the impact of the emotional quality of the parent–parent relationship on the emotional quality of the parent(s)-child relationships (Stroud et al., 2011; McCoy et al., 2013). A parent might take less care of an infant by purposely not being at home to avoid facing the other parent. On the other hand, parents with a good marital relationship are more likely to collaborate in caring for their infant, allowing the infant to experience more positive interactions with the parent(s) (Sears et al., 2016). The crossover effect, which may co-occur with the spillover effect, refers to the transfer of emotions or behavior between individuals within a subsystem rather than between subsystems or domains. In other words, a parent’s attitudes or experiences could influence the partner’s functioning with the infant (Tissot et al., 2017; Tucker et al., 2017; Miragoli et al., 2018; Pu and Rodriguez, 2021). While caring for the infant, a parent with a partner in distress (e.g., due to the presence of depressive symptoms or burnout) might present reduced availability, difficulty concentrating, and increased irritability because of worrying thoughts about the partner’s difficulties (Sutton et al., 2017). Conversely, parents who are less confident in infant care might interact with the infant with greater confidence in their gestures because they are reassured by their partners’ supportive attitude toward their parenting skills (Udry-Jørgensen et al., 2016).

To date, many studies have brought evidence of links between the quality of parent-infant (mostly mother-infant) interactions and physiological outcomes of infants’ emotion regulation in an interaction. However, no study to our knowledge has ever investigated these processes in intact biparental families, taking both the mother-child and father-child dyads into account. In the present study, we aimed to fill this gap by investigating the associations between synchrony and vagal tone within mother-infant and father-infant dyads, as well as across dyads, that is, from one dyad to the other. In particular, we examined the links between mother-infant synchrony and infants’ vagal tone during father-infant interaction, as well as the links between father-infant synchrony and infants’ vagal tone during mother-infant interaction. In line with previous research, we expected to find within-dyad associations between the variations in the quality of interactions and the infants’ physiological regulation during these interactions. Specifically, we hypothesized that high mother-infant synchrony would be linked with high infant vagal tone during the interaction with the mother. Although previous studies are scarce, we expected to find similar associations in father-infant dyads, such that higher father-infant synchrony would be linked with higher infant vagal tone during interaction with the father. As across-dyad associations have never been investigated in an empirical study to our knowledge, we formulated the exploratory hypothesis that we would find associations between high synchrony in one dyad and high infant vagal tone in the dyad, but that these associations would probably be weaker than within-dyad associations.

## 2 Method

### 2.1 Participants

The participants were a convenience sample of 84 mother–father-infant families. The mothers had a mean age of 33.75 years (*SD* = 4.00), the fathers had a mean age of 35.83 years (*SD* = 5.68; *n* = 77 due to missing data), and the infants had a mean age of 15.38 weeks (*SD* = 1.25). The infants were 44 boys and 40 girls. Mothers were mostly university graduates (54.8.0%, *n* = 70 due to missing data) with 66.7% of them employed (*n* = 56 due to missing data), 38.1% full time. Fathers were mostly university graduates (41.7%, *n* = 65 due to missing data) with 73.8% of them employed (*n* = 62 due to missing data), 63.1% full time. Mothers (*n* = 70 due to missing data) were mostly married (44%) and in a cohabiting couple (35.7%; some of them were divorced or separated from a previous relationship). Among the fathers (*n* = 65 due to missing data), 36.9% were married and 35.7% were in a cohabiting couple. A socio-economic index (IPSE) was calculated by using the formula of Genoud (2011), which is calculated based on the education level and occupation of both parents. Regarding socio-economic status, 48.8% of families belonged to the middle-upper class, 13.1% to the middle class, and 11.9% to the upper class (*n* = 72 due to missing data).

### 2.2 Procedure

In this study, we used data collected from a larger study on emotion regulation and family functioning. A midwife recruited parents around the 37th week of pregnancy at the maternity unit of the University Hospital of Geneva. We presented the objectives of the research and then provided the parents with a consent form that the interested participants signed. The midwife explained to the parents that the study’s focus was the infant’s emotions. Three months after delivery, the research team contacted and scheduled a meeting with the parents when the infant was between 3 and 4 months old. At the beginning of the meeting, the researchers reminded parents of the context and the course of the study and invited them to place the infant on a changing table. To record the measurements of the infant’s heart activity during family playtime in the study, one of the researchers placed three pediatric electrodes on the infant’s chest to record an electrocardiogram (ECG). The researcher asked the parents to interact with the infant following the family play of the Lausanne Trilogue Play paradigm (Fivaz-Depeursinge and Corboz-Warnery, 1999). In the first part, one parent played with the infant for 2 min while the other parent was outside the room. In the second part, the parents changed roles. Because in the first two parts of the play the infant interacted separately with each parent, we decided to counterbalance the order of the parts to have an equal distribution between the mothers and fathers who interacted first. Finally, in the third part, the two parents played together with the infant for 2 min. In this study, we considered only the first two parts, that is, the interactions of each parent with the infant. Before starting the interactions, the researchers indicated the position of the cameras and specified that the experiment could be interrupted at any time if the infant showed signs of excessive fatigue or distress. The researchers instructed the parents to interact, as usual, avoiding objects if possible and not to carry, pick up, or place the infant in a sitting position on the changing table to limit the recording of noise during the ECG. At the end of the interactive session, and after the removal of the electrodes from the infant, the parents were asked to fill out a form to receive online self-report questionnaires. A debriefing in the form of video feedback was offered to interested parents.

### 2.3 Measures

#### 2.3.1 Parent-infant synchrony

We assessed mother- and father-infant synchrony with the infant CARE-Index (Crittenden, 2006). The CARE-Index is an adult-infant interaction assessment that can be used from birth to 25 months. The coding system assesses global dyadic synchrony, that is, fathers’ sensitive behavior and infants’ cooperative behavior, within the context of parent-infant interactions. Scores ranged from 0 to 14, with higher scores indicating better dyadic synchrony. The total sample of 84 parent-infant interactions was coded from March 2022 to August 2022. To ensure inter-rater reliability, a random sample of 23.8% of the video recordings (20 videos in a total sample of 84) was initially coded by the first and second authors, both trained and certified as research raters in February 2022. The intraclass correlation (two-way random absolute agreement) on the synchrony scores was excellent with a coefficient of 0.982 (Koo and Li, 2016). Coders were blind to the results of the ECG analyses (see Section 2.3.2).

#### 2.3.2 Vagal tone

An ECG was recorded during baseline, mother-infant interaction, and father-infant interaction. During the 2-min baseline, the ECG was recorded while the researchers explained the instructions of the experiment, and the parents were not directly involved with the infant. Physiological data were collected with a Biopac MP160 system (Biopac Systems, Inc.) and recorded on AcqKnowledge 5.0 software (Biopac Systems, Inc.). The infants’ cardiac activity was processed on Kubios HRV v2.2 software to obtain heart rate variability measures, which reflect vagal tone. Analyses allowed us to derive the root mean square of successive differences (RMSSD), which represents the activity of the parasympathetic system and is widely considered to be a valid measure of vagal activity (Laborde et al., 2017).

### 2.4 Statistical analysis

We computed first a set of descriptive statistics for the variables under study (see Table 1). The normality test was performed by using the Shapiro–Wilk test. We also tested for bivariate correlations between the variables under study, as well as for differences depending on the order of the parts in the play through the Student’s *t*-test and the Mann–Whitney *U* test (non-parametric alternative to the Student’s *t*-test used when the samples to be compared do not have a normal distribution). Missing data analysis was conducted, as there were missing data in two control variables: fathers’ age (*n* = 7) and families’ socioeconomic status (*n* = 12). There was no missing data in the target variables. The Little’s Missing Completely at Random (MCAR) test was not significant, χ^2^ = 19.471, *df* = 20, *p* = 0.49. Which indicates that data were missing completely at random. Then, we tested for associations between the target variables and the potential control variables (sex of the infant, age of the parents, and socioeconomic status) to be included in subsequent analyses. Because of the small size of our sample, we wanted to optimize statistical power by eventually including in multivariate analyses only those control variables that would have shown significant correlations with the target variables (see Table 2 for more details). As none of the control variables showed significant correlations with the target variables, they were excluded from subsequent analyses.

**Table 1:** Descriptive statistics.

	Model 1					Model 2									
	Full sample					Mothers interacted first					Fathers interacted first				
Variable	*n*	*Min*	*Max*	*M*	*SD*	*n*	*Min*	*Max*	*M*	*SD*	*n*	*Min*	*Max*	*M*	*SD*
RMSSD M-I	84	2.95	19.80	11.20	3.48	41	3.61	19.80	11.67	3.59	43	2.95	16.90	10.76	3.36
RMSSD F-I	84	3.42	20.07	11.26	3.90	41	4.18	20.07	11.33	3.68	43	3.42	19.92	11.19	4.15
Synchrony M-I	84	1	14	8.31	3.36	41	1	14	8.71	3.53	43	2	14	7.93	3.20
Synchrony F-I	84	1	14	7.85	3.21	41	1	14	8.24	3.15	43	1	14	7.47	3.26

RMSSD, root mean square of successive differences; M, mother; F, father; I, infant.

**Table 2:** Correlation matrix for the full sample.

Full sample, *N* = 84								
Variable	1.	2.	3.	4.	5.	6.	7.	8.
1. RMSSD M-I	1							
2. RMSSD F-I	0.734**	1						
3. Synchrony M-I	0.353**	0.246*	1					
4. Synchrony F-I	0.311**	0.164	0.524**	1				
5. I sex	−0.054	−0.033	−0.111	−0.165	1			
6. M age	−0.099	−0.119	−0.122	−0.175	−0.144	1		
7. F age	0.067	0.053	−0.123	−0.156	−0.017	0.537**	1	
8. SES	0.123	0.122	0.077	−0.075	0.061	0.276*	0.278*	1

** *p* < 0.01; * *p* < 0.05; RMSSD, root mean square of successive differences; M, mother; F, father; I, infant; Sex: 1 = female, 2 = male; SES, socio-economic status: 1 = lower, 2 = lower-middle, 3 = middle, 4 = middle-upper, 5 = upper.

**Figure 1:**
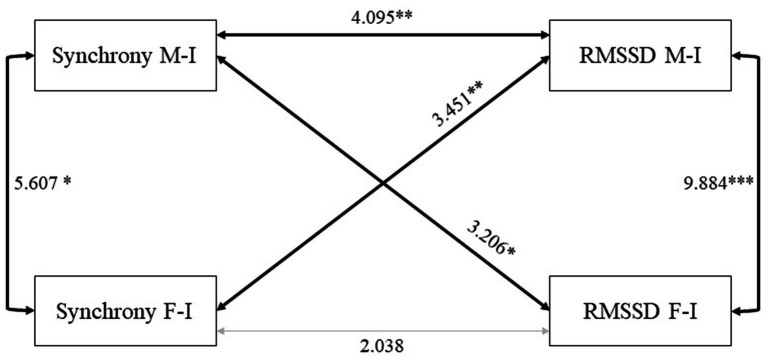
Graphical representation of the first SEM model in the full sample (= 84). *** *p* < 0.001; ** *p* < 0.01; * *p* < 0.05; RMSSD, root mean square of successive differences; M-I, mother-infant; F-I, father-infant. Structural equation modeling (SEM) shows paths between mother-infant synchrony, father-infant synchrony, and infants’ RMSSD during interactions with parents. Bold rows show significant paths between variables, and gray rows show nonsignificant paths.

To test the main hypotheses of this study, we then used structural equation modeling (SEM) techniques to test the associations between the target variables, namely, parent-infant synchrony and the infants’ RMSSD within each dyad and across one dyad to the other. In a first model (see Figure 1), we specified covariance paths between parent-infant synchrony and infants’ RMSSD to investigate their association within each dyad (see Figure 1). We refer to these covariance paths as “within-dyad” covariances. Thus, there were two within-dyad covariances in this model, one between mother-infant synchrony and infants’ vagal tone during mother-infant interactions, and one between father-infant synchrony and infants’ vagal tone during father-infant interactions. To investigate the influence across one dyad to the other, we also specified covariance paths between parent-infant synchrony in one dyad and infants’ RMSSD during the interaction in the other dyad. We refer to these covariance paths as “across-dyad” covariances. Thus, there were two across-dyad covariances in this model, one between mother-infant-synchrony and infants’ vagal tone during the interaction with the father, and one between father-infant synchrony and infants’ vagal tone during the interaction with the mother. As previous work suggested that the order of interaction (mother or father interacting first) in a family play situation may influence the parents’ behaviors during the interactions (Frascarolo et al., 2003), we conducted a multigroup analysis in a second model to test whether the order of the parts in the play influenced the study results. In this second model, the relations between the variables were specified similarly to the first model, but the model was separately estimated in two groups according to which parent interacted first (*n* = 41 families with mother playing first and *n* = 43 with father interacting first). In this model, all the parameters were left free to vary between the two groups. In order to estimate the magnitude of the differences between the two groups, we created a third nested model in which we imposed difference and equality constraints on all parameters of the model.

The first, second, and third SEM models were estimated by using a maximum likelihood with robust standard errors estimator. Because the first and second models were saturated (0 degrees of freedom), the model fit was irrelevant, as the model was perfectly fitted to the data. Information on model fit was in turn available for the third model, as it had 14 degrees of freedom. Chi-square tests and other fit indices (e.g., root mean square error of approximation [RMSEA]) were used to evaluate model fit according to the standard criteria defined by Hu and Bentler (1999). For the comparative fit index, values above 0.90 indicate a fair fit and values above 0.95 an excellent fit. For the RMSEA, values below 0.06 indicate an excellent fit and values between 0.06 and 0.08 an acceptable fit. Descriptive statistics, bivariate correlations, the Student’s *t*-test, and the Mann–Whitney *U* test were computed in IBM SPSS Statistics 27 software (IBM Corp., Armonk, NY). Mplus 7.4 (Muthén and Muthén, 2016) was used to perform SEM.

## 3 Results

### 3.1 Descriptive statistics

The mean and standard deviations of parent-infant synchrony and infants’ RMSSD during the interactions with each parent were calculated in the total sample and the two groups based on the order of the parts in the play (mother or father interacting first; see Table 1).

The Shapiro–Wilk test was performed to verify the normal distribution of the study variables, revealing that the infants’ RMSSD scores were normally distributed (with the mother, *p* = 0.783; with the father, *p* = 0.331) and that synchrony scores were not (mother-infant synchrony, *p* = 0.017; father-infant synchrony, *p* = 0.009). To investigate whether the mean scores for the target variables would vary according to the order of the parts in the play, we used the Student’s *t*-test for the infants’ RMSSD scores and the Mann–Whitney *U* test for parent-infant synchrony scores. Results revealed that the infants’ RMSSD during the interaction with the mother [*t*(82) = 1.189, *p* = 0.23] and the father [*t*(82) = 0.165, *p* = 0.86] and the synchrony scores with the mother (U: 761.000, *p* = 0.27) and the father (U: 747.000, *p* = 0.22) did not vary depending on the order of the parts in the play.

The normative values for RMSSD during infancy are predominantly rooted in 24-h ECG recordings (Massin and von Bernuth, 1997; Patural et al., 2019), which posed a challenge in their direct comparison with the 2-min segments used in the current study. However, previous investigations that focused on brief 10- or 2-min excerpts from ECG recordings during infants’ restful periods (Zeegers et al., 2017; Arce-Alvarez et al., 2019) revealed values that were either similar or slightly higher than those observed in the present sample. This minor variance might be attributed to the recording circumstances—capturing the ECG during social interactions rather than in a resting state.

### 3.2 Correlational analyses

The correlational analyses between target variables (parent-infant synchrony, and infants’ RMSSD during the interaction with each parent) and the control variables (sex of the infant, age of the parents, and socioeconomic status) were calculated in the full sample (see Table 2). The infants’ RMSSD during the interaction with the mother correlated positively and significantly with synchrony with both parents, so that, when the infants’ regulation with the mother was higher, the synchrony with both parents was also higher. The infants’ RMSSD during the interaction with the father correlated positively and significantly with mother-infant synchrony, so that, when the infants’ regulation with the father was higher, the synchrony with the mother was also higher. There was a positive and significant correlation between both synchrony scores, so the higher the synchrony with the mother, the higher the synchrony with the father. Infants’ RMSSD scores correlated positively and significantly, so that the more regulated the infants were with the mother, the more regulated they were with the father. None of the control variables showed a significant correlation with the target variables.

### 3.3 Models linking parent-infant synchrony and infants’ RMSSD

The results of the estimation of the first model (Model 1; see Figure 1 for more details) showed that the within-dyad covariance was significant and positive in the mother-infant dyad and not significant in the father-infant dyad. In other words, greater mother-infant synchrony was related to greater infant RMSSD during the interaction with the mother. The across-dyad covariance was significant and positive between mother-infant synchrony and infants’ RMSSD with the father and between father-infant synchrony and infants’ RMSSD with the mother. In other words, mother-infant synchrony was positively related to infant regulation with the father, and father-infant synchrony was positively related to infant regulation with the mother. In turn, father-infant synchrony was not related to infant RMSSD during father-infant interaction. Finally, the covariance between synchrony with the mother and father was positive and significant, such that greater synchrony related to greater synchrony, and the covariance between the infants’ RMSSDs with each parent was positive and significant so that greater regulation with one parent related to greater regulation with the other.

**Figure 2:**
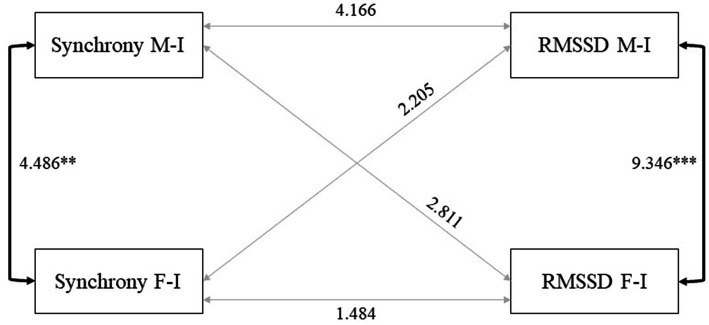
Graphical representation of the second SEM model in the group in which mothers interacted first (= 41). *** *p* < 0.001; ** *p* < 0.01; RMSSD, root mean square of successive differences; M-I, mother-infant; F-I, father-infant. Structural equation modeling (SEM) shows paths between mother-infant synchrony, father-infant synchrony, and infants’ RMSSD during interactions with parents. Bold rows show significant paths between variables, and gray rows show nonsignificant paths.

**Figure 3:**
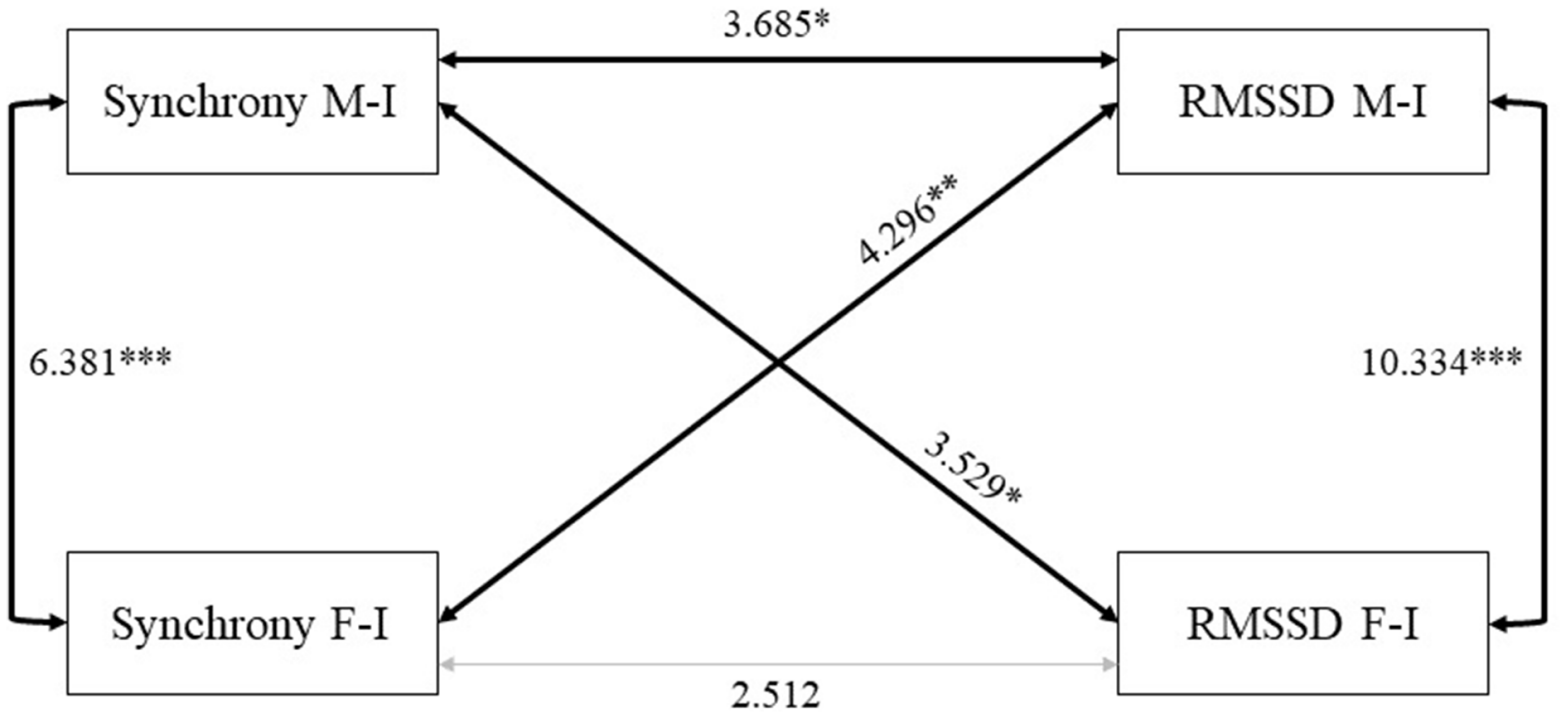
Graphical representation of the second SEM model in the group in which fathers interacted first (= 43). *** *p* < 0.001; ** *p* < 0.01; * *p* < 0.05; RMSSD, root mean square of successive differences; M-I, mother-infant; F-I, father-infant. Structural equation modeling (SEM) shows paths between mother-infant synchrony, father-infant synchrony, and infants’ RMSSD during interactions with parents. Bold rows show significant paths between variables, and gray rows show nonsignificant paths.

The results of the estimation of the second model (Model 2; see Figures 2, 3 for more details) showed that in both groups (Group 1: mothers interacted first, *n* = 41; Group 2: fathers interacted first, *n* = 43), there were three similarities: the covariance between synchrony and infants’ RMSSD in the father-infant dyads was not significant, the covariance between synchrony with the mother and father was positive and significant so that greater synchrony related to greater synchrony, and the covariance between the infants’ RMSSDs with each parent was positive and significant so that greater regulation related to greater regulation. Two differences between the groups appeared: For mothers who interacted first, the covariance between mother-infant synchrony and infants’ RMSSD with the mother was not significant. Although the covariance (i.e., unstandardized) was not significant, the correlation (i.e., standardized) was (*r* = 0.337, *p* = 0.034). In turn, this covariance was significant when mothers interacted second and fathers first. The second difference between the groups was that for mothers who interacted first, the covariance between mother-infant synchrony and infants’ RMSSD with the father was not significant. In turn, this covariance was positive and significant when fathers interacted first, such that mother-infant synchrony was related to the infant’s regulation with the father.

In the third model that aimed to test the magnitude of these differences, we imposed differences and equalities between groups on all the parameters of the second model. This model demonstrated a good fit: $\chi^{2}$ (14) = 10.802, *p* > 0.05, RMSEA = 0.000, comparative fit index = 1. The finding that the chi-square test of the third model was nonsignificant suggests that the differences between the groups, if any, were minimal, as the fit of a model specified with equality constraints on all parameters was not statistically different from a model assuming between-group differences.

## 4 Discussion

In this study, we investigated associations between interactive synchrony (an indicator of interaction quality) and infants’ vagal tone (an index of emotion regulation) during mother-infant and father-infant interactions, both within each parent-infant dyad (within-dyad) and across one dyad to the other (across-dyad).

Our hypotheses that associations exist between interactive synchrony and infants’ vagal tone within each dyad were partially confirmed. The results showed that interactive synchrony has a significant association with infants’ vagal tone within mother-infant dyads, such that variations in synchrony were related to variations in infants’ vagal tone during mother-infant interactions. Although this association was present in the whole sample (Model 1), the multigroup analyses (Model 2) revealed that this association is actually due to mother-infant dyads interacting second (i.e., after the father), as it disappears when the order of interaction is reversed. Our hypothesis that there are associations between interactive synchrony and vagal tone in infants within father-infant dyads was not confirmed in either the whole sample or subgroups based on the order of interaction. Although this lack of associations might lead to the assumption that fathers have a reduced influence on the infants’ physiological regulation of emotion, we propose looking at the family organization at 3 months in Switzerland to potentially shed light on the reasons behind the lack of associations within the father-infant dyad. At 3 months, Swiss mothers are on mandatory maternal leave and assume the role of primary caregiver, spending more time with the infant than fathers do. Fathers indeed have a shorter leave (paternity leave 2 weeks) than mothers do (maternity leave 3.5 months) and tend to work full time during the first months after their infants’ birth, reducing the opportunities for the father-infant dyads to interact (Swiss Civil Code, 2021; Federal Statistical Office, 2022). Therefore, it is plausible that the fathers in our study might have encountered limited chances for one-on-one interactions with their infants. The infrequency of these interactions could have hindered the formation of strong associations within the father-infant dyad, for which more shared time may be necessary for the development of mutual regulation. In simpler terms, increasing the duration fathers spend with their infants could have provided additional opportunities for infants to become accustomed to mutual regulation with their fathers. This, in turn, might have improved infants’ physiological responsiveness to these interactive moments, much like what is observed with mothers who are consistently present during the initial 3 months. This understanding of our results suggests that enhanced shared time during the early months may strengthen the impact of the father-infant relationship on children’s social–emotional development. To confirm the influence of shared time, future research should investigate the associations between the variables in our study by comparing groups of fathers with paternity leave of different lengths.

Moreover, the existence of significant associations across the dyads suggests that fathers’ influence may take another path, as suggested by the results. Our hypothesis that associations exist between the interactive synchrony in one dyad and infants’ vagal tone in the other dyads was indeed confirmed. The results showed that the interactive synchrony in one dyad had a significant association with infants’ vagal tone in the other dyad, such that variations in the quality of interactions in one dyad were related to variations in infants’ vagal tone during interactions in the other dyad. These associations across dyads were present in the whole sample and in the group in which fathers interacted first, revealing that they were mainly due to those father-infant dyads interacting first and those mother-infant dyads interacting second. Specifically, our results showed that father-infant synchrony was significantly associated with the infants’ RMSSD in the subsequent mother-infant interaction, whereas infants’ RMSSD during father-infant interaction was associated with mother-infant synchrony in the subsequent interaction. These results thus seem to indicate that although fathers may not have an impact on the infant’s physiological regulation during father-infant interactions, they have an indirect influence. The results across dyads in the group in which fathers interacted first suggest a way in which fathers might influence infants’ physiological regulation of emotion by influencing mother-infant synchrony and the infants’ RMSDD during mother-infant interactions. This across-dyad association in the multigroup analyses (Model 2) also suggests a potential causal relationship because the interactions occurred in sequence. Moreover, the infants’ physiological regulation of emotions during father-infant interactions might have subsequently influenced the variations in the quality of later mother-infant interactions. Further investigations are needed to assess these possible causal links.

In sum, interesting results emerged from the estimation of Model 2, in which we controlled for the influence of the order of the play, such that all within-dyad and across-dyad associations disappeared in the group of families in which mothers were asked to interact first. A speculative explanation may be proposed to explain the absence of association within mother-infant dyads when the mother interacted first. This explanation may also extend our previous explanation about the lack of associations within father-infant dyads, particularly for those father-infant dyads that interacted first. In our study, just before the start of the two parts of the play, the infants were barely stimulated by the parents engaged in listening to the researchers’ instructions. Once alone with the first interacting parent, the infants had to “tune in” to the parents’ request to interact. This moment of attunement may have delayed the establishment of coregulatory processes within the dyads and their associations with the physiological patterns of the infant, regardless of the dyads’ increased habit of interaction at 3 months. Further investigation is required to delve into our speculative explanation, as well as to understand the reasons behind the absence of associations across dyads in the group in which fathers interacted second. Gaining a more comprehensive understanding of how the order of interaction affects parent-infant interactions could yield profound insights into the influences molding infant physiological regulation of emotion. In turn, this broader understanding will enhance the interpretation of the results of this study.

This study has some limitations. Most of the participants belonged to the middle-upper socio-economic class in the Swiss population and were university graduates. Furthermore, most of the study participants lived in a heterosexual two-parent family, so we had to limit our analysis to this group. Our results may therefore be different in other types of families. Although a global assessment of the interactive synchrony considers the behavioral patterns within mother- and father-infant dyads, it does not allow for the investigation of the association between specific interactive synchrony behaviors (e.g., sharing of smiles, the direction of gaze toward the other partner, demonstration of readiness for interaction, and vocalizations) and changes in vagal tone. The systemic nature of emotion regulation involves physiological, affective, and social mechanisms (Barrett, 2017; Thompson, 2019; Pruessner et al., 2020). Thus, although vagal tone is often used in studies as the main indicator of emotion regulation, other indicators could have captured the contextual and extrinsic factors crucial for infant emotion regulation. In addition to vagal tone, future studies should also consider the observed behaviors of emotion regulation during interaction so that the findings of this study can be further confirmed.

Our study is the first to consider the association between the quality of interactions and the vagal tone of 3-month-old infants, both within each parent-infant dyad and across one dyad to the other during subsequent interactions. Notwithstanding its limitations, our study shows the existence of associations between interactive processes and infants’ physiological regulation of emotions within the mother-infant dyad and across dyads in a family. The associations across dyads provide evidence that the quality of father-infant interactions has a crucial influence on family relational dynamics, with consequences for the early physiological regulation of infant emotions. However, the significance of the associations may vary when controlling for the order of interaction, demonstrating that interactive processes within and beyond the dyad are sensitive to contextual factors and interdependencies between family members. Future research with a systemic perspective of family relationships is needed to investigate the complex family influences on the socio-emotional development of the infant.

## Data availability statement

The datasets presented in this study can be found in online repositories. The names of the repository/repositories and accession number(s) can be found at: https://doi.org/10/gsrmqw.

## Ethics statement

The studies involving humans were approved by Ethical Committee of the State of Geneva. The studies were conducted in accordance with the local legislation and institutional requirements. Written informed consent for participation in this study was obtained from the participants.

## Author contributions

NP: Conceptualization, Data curation, Formal analysis, Investigation, Methodology, Software, Validation, Visualization, Writing – original draft, Writing – review & editing. NF: Conceptualization, Data curation, Funding acquisition, Project administration, Resources, Supervision, Validation, Visualization, Writing – review & editing. VR: Data curation, Formal analysis, Investigation, Software, Visualization, Writing – review & editing. ME: Funding acquisition, Resources, Writing – review & editing. CR: Funding acquisition, Resources, Writing – review & editing. HT: Conceptualization, Data curation, Formal analysis, Funding acquisition, Methodology, Project administration, Resources, Software, Supervision, Validation, Visualization, Writing – review & editing.
